# An Extremely Rare Metastatic Prostate Tumor From Rectal Cancer With Characteristic MRI Findings Due to Necrosis

**DOI:** 10.7759/cureus.82191

**Published:** 2025-04-13

**Authors:** Sohei Iwagami, Shoji Oura, Haruka Miyai, Naoki Kataoka, Masaya Nishihata

**Affiliations:** 1 Urology, Kishiwada Tokushukai Hospital, Kishiwada, JPN; 2 Surgery, Kishiwada Tokushukai Hospital, Kishiwada, JPN

**Keywords:** garland necrosis, metastatic prostate tumors, mri, prostate cancer, rectal cancer

## Abstract

Colorectal carcinomas rarely metastasize to the prostate. Early detection of metastatic prostate tumors can lead to possible cure with less invasive treatment. A 71-year-old man was diagnosed with rectal cancer and underwent surgery for it. The postoperative pathological study showed atypical cells growing mainly cribriform and some large necrosis in the tubule-forming structures. After favorable control of the recurrent lesion for 30 months, the MRI showed the right lobe tumor with low signals on diffusion-weighted images. CT clarified the enlargement of the right lobe mass in the prostate four years after the surgery. The patient, therefore, underwent total pelvic exenteration, including prostatectomy. The postoperative pathological study showed metastatic adenocarcinoma from rectal cancer with massive garland necrosis. Diagnostic physicians should note that rectal cancer can, though extremely rarely, metastasize to the prostate and that the presence of garland necrosis can make image diagnosis difficult.

## Introduction

Colorectal cancer is the leading cause of cancer morbidity among men in developed countries including the United States and Japan. Colorectal cancer metastasizes, in order of frequency, to the liver, lungs, lymph nodes, and peritoneum [1]. Regarding the treatment of lung and liver metastases from colorectal cancer, often observed in clinical practice, optimal treatments, including surgery, have already been established.

The prostate is an extremely rare target organ for malignant diseases to metastasize. Especially among various solid malignancies, rectal cancer develops very closely to the prostate and has various common venous and lymphatic channels from the prostate. However, it is extremely rare for rectal cancer to directly invade or metastasize to the prostate [2]. Therefore, prostate invasion from rectal cancer and metastatic prostate tumors are very rarely considered in the differential diagnosis of prostate tumors.

Low incidence of prostate invasion and metastatic prostate tumors has hindered the establishment of optimal treatments for them. Total pelvic exenteration, a highly invasive therapeutic option for the patient, allows at least macroscopic complete removal of the prostate tumor in the vast majority of cases [3]. In order to get a cure with less invasive treatment, it is essential to diagnose invasion or metastasis to the prostate at an earlier stage. We present a case in which the diagnosis was slightly delayed due to atypical MRI findings. Fortunately, the patient did not suffer any major disadvantages, but we believe that similar metastatic cases could be disadvantageous.

We herein report a metastatic prostate tumor of rectal cancer that co-existed with primary prostate cancer, which presented extremely atypical images.

## Case presentation

**Figure 1:**
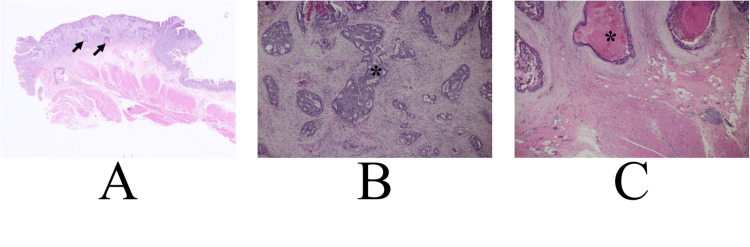
Pathological findings of the rectal cancer A. The image shows a moderately differentiated tubular adenocarcinoma invading the submucosa. B. The low-magnified view shows that well-differentiated cancer cells spread widely in the rectal mucosa and submucosa and focal garland necrosis in the tubule-forming structures (arrows). C. The magnified view shows that cancer cells formed tubule-forming structures and necrotic tissue within some of them. The image shows garland necrosis: abundant intraluminal dirty necrosis (asterisk).

A 71-year-old man was referred to our hospital for a detailed examination of constipation. Colonoscopy and staging examination clarified that rectal cancer without distant metastasis caused the constipation. The patient, therefore, underwent low anterior resection and regional node dissection. Postoperative pathological study showed that the rectal cancer had atypical cells growing mainly in a cribriform fashion, submucosal invasion of them with garland necrosis, no positive margins, and no lymph node metastasis (Figure 1).

**Figure 2:**
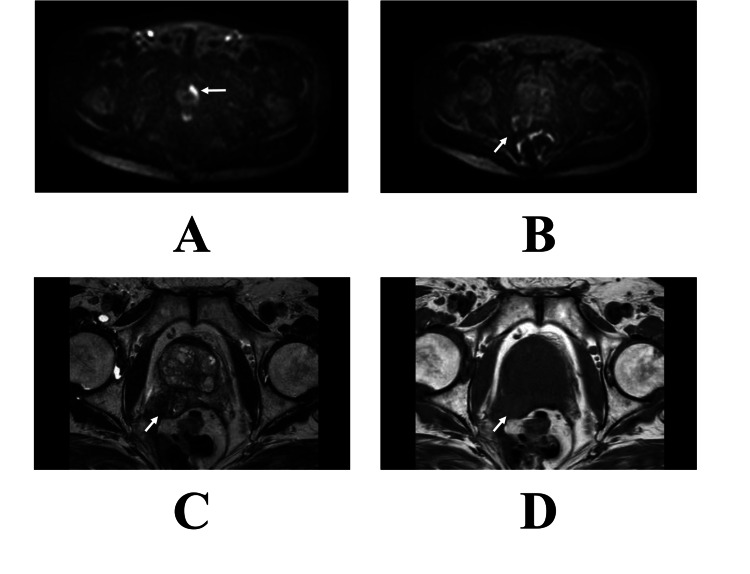
MRI findings A. MRI shows high signals on diffusion-weighted images in the left transition zone (arrow). B. MRI shows predominant low signals on diffusion-weighted images in the right periphery zone (arrow). C. MRI shows a presumed metastatic mass with low signals on T1-weighted coronal images in the right periphery zone (arrow). D. MRI shows low signals on T2-weighted images, presumably corresponding to the metastatic tumor (arrow). MRI, magnetic resonance imaging

The patient, therefore, had received no adjuvant therapies. Follow-up computed tomography (CT) showed a presumed lymph node metastasis around the obturator foramen one year after surgery. The idea that surgery might cause serious complications to the patient made us treat the patient with a non-surgical salvage therapy. The patient, therefore, underwent intensity-modulated radiotherapy to the swollen lymph node, leading to regression of the target focus. Around 42 months after surgery, i.e., 30 months after salvage radiotherapy, the patient developed urinary dysfunction with an elevated prostate-specific antigen (PSA) level of 6.87 ng/mL. MRI of a prostate mass in the left transition zone showed low signals on T2-weighted images and high signals on diffusion-weighted images. MRI of the prostate mass, 20 mm in size, in the right periphery zone showed high signals both on T1- and T2-weighted images and low signals on diffusion-weighted images (Figure 2).

**Figure 3:**
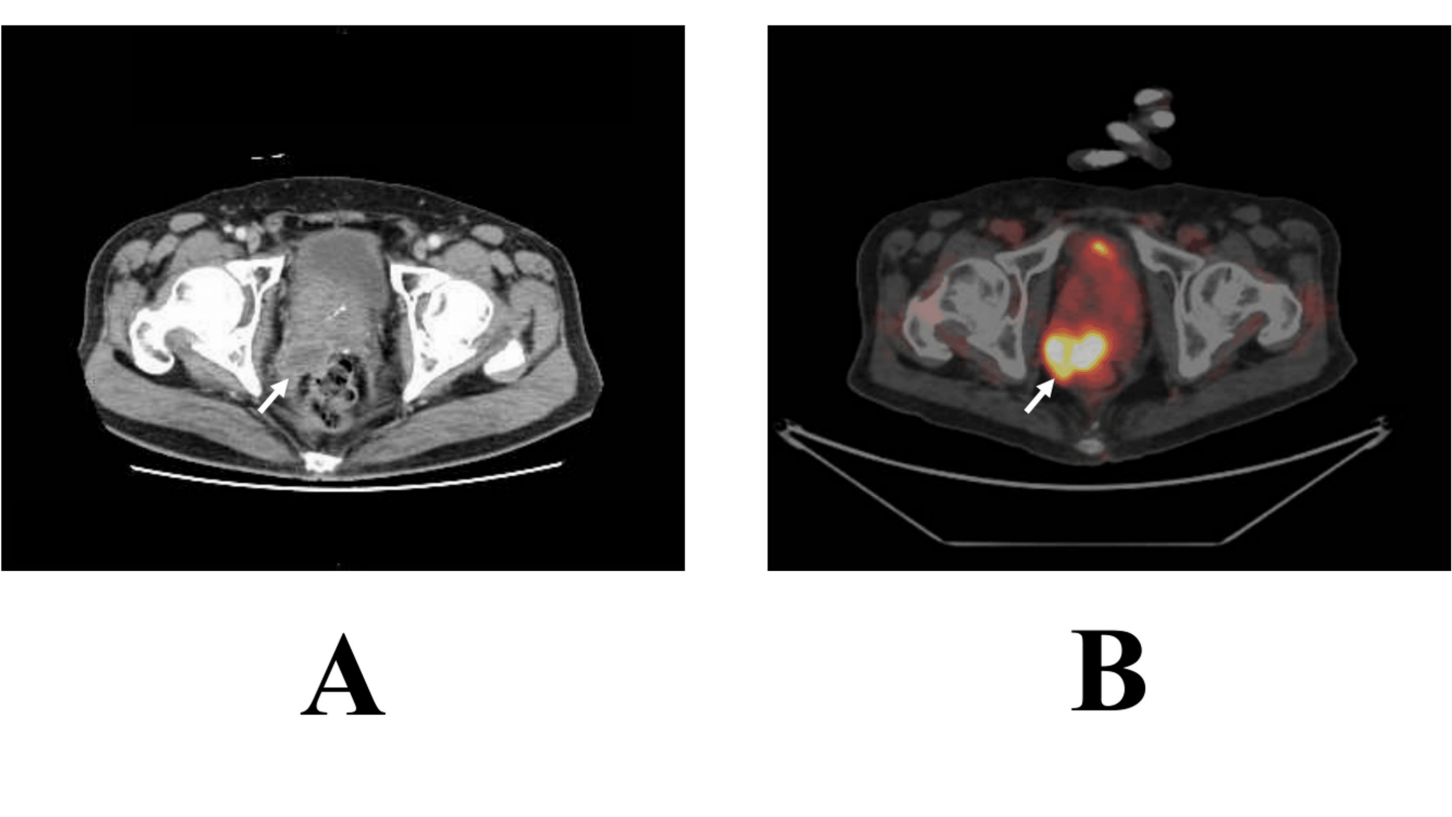
PET and CT findings A. The presumed metastatic tumor had a ring enhancement (arrow). B. PET showed a maximal standardized uptake value of 8.9 g/mL (arrow). PET, positron emission tomography; CT, computed tomography

Pathological evaluation of the transperineal prostate biopsy specimen from the left lobe showed Gleason score 6 adenocarcinoma cells. The patient, therefore, started receiving hormone therapy for prostate cancer, and PSA levels continued to decrease. Around 48 months after surgery, contrast-enhanced computed tomography (CT) showed enlargement of the right lobe tumor and elevated tumor markers such as CEA and CA19-9, highly suggesting the recurrence of the rectal cancer (Figure 3). The patient, therefore, received chemotherapy under the tentative diagnosis of rectal cancer recurrence. Despite no progression with the chemotherapy, treatment-induced renal failure made it difficult for the patient to continue the chemotherapy. Positron emission tomography (PET)/CT of the presumed metastatic tumor showed a maximal standardized uptake value (SUV) of 8.9 g/mL (Figure 3).

**Figure 4:**
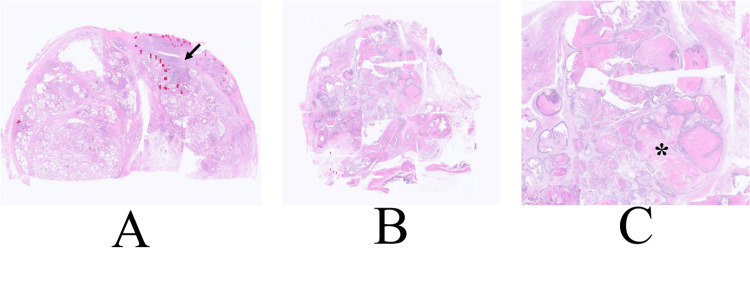
Pathological findings A. Prostate cancer was observed in the very limited areas of the left lobe of the prostate (arrow). B. Low magnified view shows that the right peripheral zone of the prostate was occupied with rectal cancer cells with garland necrosis. C. Magnified view shows that the tumor consisted mainly of garland necrosis encompassed by thin cancer cell layers (asterisk).

The patient, therefore, inevitably underwent total pelvic exenteration in order to get a possible cure. In addition to the small prostate cancer, postoperative pathological evaluation showed atypical cells growing cribriform and papillary fashions with massive garland necrosis (Figure 4).

The patient recovered uneventfully, was discharged on the 24th day after surgery, and has been well for five months.

## Discussion

The mechanisms of solid cancer metastasis include hematogenous, lymphatic, transluminal, direct invasive, and disseminated spread [2]. Transluminal, direct invasive, and disseminated spread could be easily ruled out by the pathological findings in this case. Metastasis to the prostate, therefore, was considered to be caused by hematogenous or lymphatic spread. The rectum and the prostate share venous and lymphatic pathways anatomically [4], making it difficult for us to precisely judge the true metastatic mechanism.

Both rectal cancer surgery and radiation therapy to the recurrent lesion could have affected this prostate metastasis. Rectal cancer surgery naturally has a significant effect on the venous or lymphatic flow in the pelvis, possibly having caused the prostatic metastasis in this case. However, although numerous patients undergo surgery for rectal cancer, prostatic metastasis of rectal cancer itself is extremely rare. Radiation therapy after surgery, therefore, could have been highly involved in the formation of this metastatic prostate tumor [5-7].

Garland necrosis, i.e., intraluminal "dirty" necrosis, is often found in colorectal adenocarcinomas and, when found in metastatic lesions, helps identify the primary tumor [8,9]. It is well known that malignant tumors generally have heterogeneity. In addition, it is common knowledge among clinicians that highly malignant components much often metastasize to distant organs than less aggressive components. In other words, poorly differentiated components or components with some kind of necrosis including garland necrosis are more likely to distantly metastasize than tubule-forming components [10]. This common knowledge well explains why garland necrosis was seen only in a small proportion of rectal cancer tissue but was seen in large amounts in metastatic prostate tumor.

MRI of the metastatic prostate tumor showed low signals all on T1-, T2-, and diffusion-weighted images. These findings were well explained by no or sparse presence of protons in the metastatic prostate tumor [11]. Regardless of the type of necrosis, immediately after its occurrence, it generally contains a large number of protons, i.e., watery. The necrotic areas of garland necrosis are initially watery but lose protons over time. Therefore, despite the presence of viable cells in the metastatic prostate tumor, MRI showed low signals on both T1- and T2-weighted images due to the presence of large amount of garland necrosis. Proton depletion also well explains the low signals on diffusion-weighted images in this case.

Limitations of this report are the small number of cases due to the rarity of this disease and the lack of sufficient data to prove the hypothesis due to the few reports of metastases with necrosis. We believe that the present discussion is well-considered and deserves recognition. In addition, the lack of data may delay early detection and treatment, and it is essential to continue accumulating such data. Our discussion will greatly help clinicians when they encounter similar cases in the future.

## Conclusions

Rectal cancer extremely rarely develops metastasis to the prostate despite the anatomically closest location between them. On the other hand, rectal cancer can have garland necrosis and an enhanced presence of it in metastatic lesions. The presence of garland necrosis may adversely affect the assessment of tumor viability. Physicians, therefore, should note that the presence of garland necrosis in colorectal cancer may lead to underestimation in the assessment of presumed metastatic foci.
